# Effect modification by vitamin D receptor genetic polymorphisms in the association between cumulative lead exposure and pulse pressure: a longitudinal study

**DOI:** 10.1186/1476-069X-14-5

**Published:** 2015-01-13

**Authors:** Min A Jhun, Howard Hu, Joel Schwartz, Marc G Weisskopf, Linda H Nie, David Sparrow, Pantel S Vokonas, Sung Kyun Park

**Affiliations:** Department of Epidemiology, University of Michigan School of Public Health, Ann Arbor, MI USA; Dalla Lana School of Public Health, University of Toronto, Toronto, ON Canada; Department of Environmental Health, Harvard School of Public Health, Boston, MA USA; School of Health Sciences, Purdue University, West Lafayette, IN USA; Veterans Affairs Boston Healthcare System and Boston University School of Medicine & Public Health, Boston, MA USA

**Keywords:** Lead, Vitamin D receptor, Gene by environmental interaction, A longitudinal study

## Abstract

**Background:**

Although the association between lead and cardiovascular disease is well established, potential mechanisms are still poorly understood. Calcium metabolism plays a role in lead toxicity and thus, vitamin D receptor (*VDR*) polymorphisms have been suggested to modulate the association between lead and health outcomes. We investigated effect modification by *VDR* genetic polymorphisms in the association between cumulative lead exposure and pulse pressure, a marker of arterial stiffness.

**Methods:**

We examined 727 participants (3,100 observations from follow-ups from 1991 to 2011) from the Normative Aging Study (NAS), a longitudinal study of aging. Tibia and patella bone lead levels were measured using K-x-ray fluorescence. Four single nucleotide polymorphisms (SNPs) in the *VDR* gene, *Bsm1*, *Taq1*, *Apa1*, and *Fok1*, were genotyped. Linear mixed effects models with random intercepts were implemented to take into account repeated measurements.

**Results:**

Adjusting for potential confounders, pulse pressure was 2.5 mmHg (95% CI: 0.4-4.7) and 1.9 mmHg (95% CI: 0.1-3.8) greater per interquartile range (IQR) increase in tibia lead (15 μg/g) and patella lead (20 μg/g), respectively, in those with at least one minor frequency allele in *Bsm1* compared with those with major frequency allele homozygotes. The observed interaction effect between bone lead and the *Bsm1* genotype persists over time during the follow-up. Similar results were observed in effect modification by *Taq1*.

**Conclusions:**

This study suggests that subjects with the minor frequency alleles of *VDR Bsm1* or *Taq1* may be more susceptible to cumulative lead exposure-related elevated pulse pressure.

**Electronic supplementary material:**

The online version of this article (doi:10.1186/1476-069X-14-5) contains supplementary material, which is available to authorized users.

## Background

Lead (Pb) is known to increase the risk of cardiovascular diseases (CVD). A recent expert review by the National Toxicology Program (NTP) concluded that there is sufficient evidence that long-term cumulative exposure, even at low level exposure, which can be assessed by bone lead levels used in our study, is associated with elevated blood pressure [1]. Several studies have identified a positive association between lead exposure and high blood pressure, which is a major risk factor for CVD. Martin et al. [2] found an association between blood lead levels and increases in blood pressure, as well as an association between tibia bone lead levels and hypertension in the Baltimore Memory Study. Cheng et al. [3] reported an increased risk of incident hypertension with higher levels of lead in tibia and patella bones in the Normative Aging Study (NAS). Another study, which used the NAS data, found an association between high lead levels in bone and blood and hypertension among subjects with low dietary calcium intake [4]. Blood lead levels have also been associated with increased risk of pregnancy-induced hypertension [5]. The association between lead exposure and the risk of hypertension in pregnant women was also demonstrated even in those with blood lead levels less than 2 μg/dL [6]. Lead exposure may also induce endothelial injury and atherosclerosis [7–9]. The study by Perlstein et al. [10] suggests that lead accumulation may contribute to the increase in pulse pressure, a measure of arterial stiffness, and with clinical cardiovascular events. Zhang et al. [11] also reported a deleterious impact of cumulative lead on pulse pressure with effect modification by hemochromatosis genetic polymorphism in the NAS.

Vitamin D receptor (VDR) is involved in lead absorption and accumulation [12]. Vitamin D plays an important role in calcium metabolism, which is shared by lead [13]. Calcitriol, an active hormonal form of vitamin D, stimulates increased calcium absorption from the gut during calcium deficiency. Calcitriol also stimulates the expression of genes engaged in absorption of calcium in the intestine. The effects of vitamin D and calcitriol are mediated by their interaction with the VDR. The complex of calcitriol and VDR acts as a transcription factor regulating the gene expression of calcium-binding receptors. Because lead is a divalent cation, lead competes with calcium to bind to calcium-binding receptors [14]. When calcium levels are low, the calcium-binding protein may bind lead instead of calcium, resulting in elevated absorption of lead [15]. The *VDR* genetic variants have been identified as potential genetic factors that can influence the absorption, retention and accumulation of lead in the human body [12]. Schwartz et al. [16] examined former organolead manufacturing workers and found that *Bsm1* variant on the *VDR* gene modifies the association between age and tibia bone lead levels. In this study, the cumulative level of lead in bone and the rate of reabsorption and excretion of lead over time were higher for those with the *VDR Bsm1* variant. This study suggests that the *VDR* genetic variant may play a role in susceptibility to lead accumulation.

Vitamin D and VDR are involved in arterial stiffness and arterial aging [17, 18]. Vitamin D has been demonstrated to regulate endothelial nitric oxide synthase and arterial stiffness in a mouse study [19]. Lower serum vitamin D_3_ levels are known to be associated with hypertension [20]. VDR is also involved in the renin-angiotensin system, cell proliferation and differentiation, anti-inflammation, and anti-fibrosis [21]. The effect of VDR on immune response and inflammation has been related to atherosclerosis [22].

There have been studies examining effect modification of lead by *VDR* in relation to several diseases [23, 24]. However, effect modification by *VDR* genotype in the association between lead and subclinical CVD measures, including arterial stiffness in longitudinal settings, has not yet been examined. In this study, we investigate effect modification by the *VDR* gene in the association between cumulative lead exposure measured by bone lead levels and pulse pressure, a marker of arterial stiffness.

## Methods

### Study population

The NAS is a longitudinal study of the aging process established by the Veterans Administration in 1963 at the VA Outpatient Clinic in Boston, Massachusetts. The participants were 2,280 mostly White men aged 21 to 80 years with no past or present known chronic conditions (heart disease, cancer, recurrent asthma, sinusitis, bronchitis, diabetes, gout, peptic ulcer, or hypertension) [25]. The NAS followed up on the participants every 3 to 5 years.

The NAS participants were invited to obtain bone lead measurements between 1991 and 1999 at the Ambulatory Clinical Research Center of the Brigham and Women’s Hospital in Boston, Massachusetts (N = 866). Of the participants with bone lead measurements, nine participants were excluded due to unreliable bone lead measurements (detailed description in the lead exposure section). Of 857 participants, 727 participants were successfully genotyped for at least one single nucleotide polymorphisms (SNP) on the *VDR* gene including *Bsm1* (rs1544410), *Taq1* (rs731236), *Apa1* (rs7975232), and *Fok1* (rs10735810). The present analysis includes pulse pressure measured at the time of bone lead measurement (baseline, 1991–1999) and follow-up data through June 22, 2011. A total of 3,100 observations (727 participants) were used in this study. Each participating institute’s institutional review board approved this study and written informed consent was collected from each participant.

### Blood pressure

The participants visited the study center in the morning. The participants were asked not to smoke or drink for at least 12 hours before a visit. Seating systolic blood pressure (SBP) and fifth-phase diastolic blood pressure (DBP) were measured to the nearest 2 mmHg. Blood pressures were measured in the left arm and then in the right arm with a standard mercury sphygmomanometer with a 14-cm cuff. The mean measurements of the left and right arms were used in this study. Pulse pressure was calculated as the difference between SBP and DBP.

### Lead exposure

Bone lead measurement is used as an index of cumulative lead exposure levels. Tibia (the mid-shaft of the left tibia, cortical) and patella (the left patella, trabecular) bone lead levels were measured using a K-x-ray fluorescence instrument (KXRF) (ABIOMED, Danvers, MA) [26]. Participants with higher than 10 μg/g or 15 μg/g uncertainty of tibia or patella bone lead levels, respectively, (reflecting precision of the estimates) were excluded (n = 9). More details were published in a previous study [27].

### Genotyping

Multiplex polymerase chain reaction assays were designed with Sequenom Spectro DESIGNER software (Sequenon, Inc, San Diego, CA) by inputting sequences containing the SNP site and 100 base pairs of flanking sequence on either side of the SNP. In the *VDR* gene, four SNP including *Bsm1* (rs1544410), *Taq1* (rs731236), *Apa1* (rs7975232), and *Fok1* (rs10735810) were genotyped. More details on genotyping were provided in a previous study [27].

### Statistical analysis

When the data were investigated without assuming any inheritance models for the SNPs, we observed that a dominant inheritance model (in terms of minor frequency allele) fits the data best (data not shown). For dominant model, genotype was coded as 0 if a subject had no minor frequency allele (ancestral type), or was coded as 1 if a subject had one or two minor frequency alleles (variant type). The study population was partitioned based on the genotypes and compared with regard to baseline characteristics. For each of the four SNPs, the Hardy-Weinberg Equilibrium was checked for an evidence of inbreeding, population stratification, and problems in genotyping.

To account for the repeated measurements on pulse pressure and covariates, linear mixed effects models with random intercepts were implemented. We decomposed age at examination into age-at-baseline and time-since-baseline to capture the baseline age effect as well as the longitudinal aging effect [28]. The main effects of bone lead levels and *VDR* genotype, and their interaction term were fitted adjusting for time-since-baseline and an interaction term between time-since-baseline and bone lead levels in addition to the following covariates: age at baseline, race (White or not), body mass index (BMI), smoking (pack-years), alcohol intake (two or more drinks/day; Yes/No), calcium intake from food (calcium (mg)/day), diabetes status (Yes/No), antihypertensive medication status (Yes/No), family history of hypertension (Yes/No), education (less than high school, high school, some college, or four year college or more), and age at baseline by *VDR* genotype interaction. The interaction between time-since-baseline and bone lead would capture different trajectories of pulse pressure over time in relation to bone lead levels. A three-way interaction among time-since-baseline, bone lead and *VDR* genetic polymorphism was initially considered but not included in our final model because it was almost null, suggesting that the lead by *VDR* genotype interaction did not change over time and that the lead by time-since-baseline interaction did not differ between the *VDR* genotypes. The mixed model we used is described as follows:


where *Y*_*ij*_ is pulse pressure of subject *i* at time *j*, *β*_0_ is a fixed intercept, *β*_1_-*β*_5_ are fixed coefficients representing the estimated effects of each following term, *u*_*i*_ is the random intercept that reflects unexplained subject to subject heterogeneity that induces correlation among observations from the same subject, and *ε*_*ij*_ is a random error.

To interpret longitudinal associations of pulse pressure with tibia levels, we computed the predicted values of pulse pressure from the model with the continuous tibia lead variable using the tibia bone lead values at the 25th percentile and the 75th percentile of the distribution at the baseline (time = 0) and after 10 years of follow-up (time = 10) for the ancestral type and variant type, with all covariates held constant at the mean (continuous variables) or zero (categorical variables)

## Results

All the four SNPs on the *VDR* gene, *Bsm1*, *Taq1*, *Apa1*, and *Fok1*, are common SNPs with a minor allele frequency range of 0.37 to 0.45 (Additional file 1: Table S1). All SNPs were in Hardy-Weinberg equilibrium. The *Bsm1* is in a strong linkage disequilibrium (r^2^ = 0.92) with *Taq1* in this study population but not with *Apa1* (r^2^ = 0.54) or *Fok1* (r^2^ = 0.001). Genotypes missing rates were low (range: 1 - 6%). Among 727 participants, 442 participants (61%) had at least one copy of the *Bsm1* minor frequency allele (bb or Bb, variant type) and 238 participants (33%) were homozygous for the major frequency allele (BB, ancestral type). Forty seven subjects (6%) had a missing *Bsm1* genotype.

The mean age at baseline of the study population was 66 years (range: 48–93 years). The participants were followed for up to 20 years. The median follow-up period was longer for subjects who had at least one copy of the *Bsm1* minor frequency allele than for subjects who did not have the minor frequency allele (12 years vs. 9 years, Table 1). The number of follow-up examinations ranged from 1 to 8 with a median of 4. More than half of the participants were examined at least 5 times over 10 years.

**Table 1:** **Characteristics of study population by the number of genetic variants at baseline**

Characteristics		Missing	No. of minor frequency allele on ***Bsm1***		
	All	***Bsm1***	0	1	2
Number of subjects	727	47 (6%)	238 (33%)	316 (43%)	126 (17%)
Follow-up (years, mean ± SD)	10.6 ± 5.5	9.6 ± 6.2	9.9 ± 5.8	11.1 ± 5.2	11.3 ± 5.1
No. of follow-up exams (median (Q1-Q3))	4 (3–6)	4 (2–6)	4 (2–6)	4.5 (3–6)	4.5 (3–6)
***Continuous variables (mean ± SD)***					
Age at baseline (years)	66.4 ± 7.2	66.8 ± 8.9	66.7 ± 7.1	66.1 ± 7.1	66.6 ± 6.9
Height (m)	1.7 ± 0.1	1.7 ± 0.1	1.7 ± 0.1	1.7 ± 0.1	1.7 ± 0.1
Waist circumference (cm)	984.3 ± 94.1	97.6 ± 9.2	98.9 ± 9.4	985.1 ± 92.8	976.4 ± 98.3
Body Mass Index (kg/m^2^)	27.9 ± 3.7	27.4 ± 3.5	28.2 ± 3.9	27.9 ± 3.5	27.6 ± 3.7
High-density lipoprotein (HDL) cholesterol (mg/dL)	47.8 ± 12.4	47.4 ± 13.2	48.7 ± 12.3	47.5 ± 12.7	47.3 ± 11.5
Total cholesterol-to-HDL ratio	5.1 ± 1.5	5.3 ± 1.9	4.9 ± 1.3	5.1 ± 1.6	5.0 ± 1.3
Smoking (pack-years)	21.0 ± 25.1	21.5 ± 24.9	24.5 ± 28.2	19.6 ± 23.5	17.7 ± 22.2
Calcium intake (mg/day)	806 ± 404	916 ± 425	780 ± 388	817 ± 401	789 ± 427
Sodium intake (mg/day)	3855 ± 1841	4022 ± 1673	3770 ± 1700	3970 ± 2093	3658 ± 1407
Potassium intake (mg/day)	3363 ± 1386	3895 ± 1413	3212 ± 1232	3467 ± 1568	3191 ± 1067
Total calories intake (kcal/day)	1992 ± 637	2208 ± 770	1936 ± 651	2015 ± 615	1955 ± 599
Physical activity (kcal/week)	2002 ± 1788	1963 ± 1743	1879 ± 1755	2131 ± 1833	1921 ± 1752
Systolic blood pressure (mmHg)	136.0 ± 17.3	135.5 ± 18.2	137.6 ± 17.9	135.2 ± 17.8	135.3 ± 14.4
Diastolic blood pressure (mmHg)	81.7 ± 9.6	80.7 ± 10.6	83.2 ± 9.6	81.3 ± 9.3	80.3 ± 9.4
Pulse pressure (mmHg)	54.3 ± 14.7	54.8 ± 15.9	54.5 ± 14.5	53.8 ± 15.3	55.0 ± 12.8
Tibia lead level (μg/g)	21.2 ± 13.2	20.4 ± 14.5	22.2 ± 13.3	20.6 ± 13.2	21.2 ± 12.8
Patella lead level (μg/g)	30.5 ± 19.3	27.3 ± 15.9	31.9 ± 21.4	29.3 ± 17.8	32.0 ± 19.9
Blood lead level (μg/dL)	5.9 ± 3.9	6.0 ± 4.4	6.5 ± 4.1	5.7 ± 3.7	5.6 ± 3.7
***Categorical variables (n(%))***					
Race (white or not)	703 (97%)	45 (96%)	227 (95%)	310 (96%)	121 (96%)
Alcohol (two or more drinks/day)	148 (20%)	7 (15%)	42 (18%)	76 (24%)	23 (18%)
Diabetes (diagnosed or taking medication)	94 (13%)	7 (15%)	28 (12%)	39 (12%)	20 (16%)
Antihypertensive medication	128 (18%)	8 (17%)	44 (18%)	54 (17%)	22 (17%)
Family history of hypertension	438 (60%)	21 (45%)	141 (59%)	201 (64%)	75 (60%)
Education: Less than high school	70 (10%)	1 (2%)	31 (13%)	19 (6%)	19 (15%)
Complete high school	248 (34%)	14 (30%)	87 (37%)	106 (34%)	41 (33%)
Some college	179 (25%)	15 (32%)	53 (22%)	81 (26%)	30 (24%)
College or more	204 (28%)	16 (34%)	62 (26%)	97 (31%)	29 (23%)

The subjects with a missing *Bsm1* genotype were not substantially different from the remaining subjects for the baseline characteristics including age, race, BMI, SBP, DBP, pulse pressure, and blood pressure control medication status (Table 1). Tibia and patella bone lead levels, and the proportion of subjects with a family history of hypertension were slightly lower for those with the missing genotype. In contrast, calcium, sodium, potassium, and alcohol intake were slightly higher among subjects with the missing *Bsm1* genotype. The baseline characteristics were similar between participants with ancestral type and those with variant type. Participants with ancestral type smoked more, had a higher DBP, and had slightly lower prevalence of whites.

In longitudinal analyses, the two-way interaction between tibia bone lead levels and time-since-baseline was significant (estimate of the interaction term between tibia bone lead and time-since-baseline in the *Bsm1* model: -0.013, 95% CI = (-0.021, -0.005), p = 0.0008; in the *Taq1* model: -0.014, 95% CI = (-0.021, -0.006), p = 0.0002), suggesting that the association between bone lead levels and pulse pressure diminished over time. On the other hand, the regression coefficient for the three-way interaction term of *VDR* genotype, tibia bone lead and time-since-baseline was close to zero (estimate of the three-way interaction term in *Bsm1*: 0.005, 95% CI = (-0.004, 0.014), p = 0.28; in *Taq1*: 0.004, 95% CI = (-0.005, 0.013), p = 0.37). This suggests that the difference in the association between bone lead and pulse pressure by *VDR* genotype was constant during the follow-up.

Table 2 shows the estimated regression coefficients and 95% confidence intervals for the association between bone lead and pulse pressure by *VDR* genotype at baseline (i.e., time is fixed at zero). With an interquartile range (IQR) increase in tibia lead (15 μg/g), pulse pressure was 2.5 mmHg (95% CI: 0.4-4.7) greater for the participants with variant type on *Bsm1* compared with the participants with ancestral type (Table 2). With an IQR increase in patella lead (20 μg/g), pulse pressure was 1.9 mmHg (95% CI: 0.1-3.8) greater for the participants with at least one copy of the minor frequency allele in *Bsm1* compared with the participants without the minor frequency allele (Table 3). Similar results were found for *Taq1*. The interaction effect was relatively smaller for *Apa1* and *Fok1*.

**Table 2:** **Adjusted changes in pulse pressure (mmHg) with an IQR (15 μg/g) increase in tibia lead levels**

SNP	N	Ancestral vs. Variant	Interaction term	
		β (95% CI)*	β (95% CI)*	P
***Bsm1***	816	Ancestral 0.1 (-1.8, 1.9)	2.5 (0.4, 4.7)	0.02
	1626	Variant 2.6 (1.2, 4.0)		
***Taq1***	827	Ancestral 0.4 (-1.4, 2.2)	2.0 (-0.1, 4.1)	0.06
	1760	Variant 2.4 (1.1, 3.8)		
***Apa1***	795	Ancestral 1.5 (-0.4, 3.4)	0.3 (-1.9, 2.4)	0.81
	1787	Variant 1.8 (0.4, 3.1)		
***Fok1***	974	Ancestral 2.1 (0.5, 3.7)	-0.02 (-2.0, 2.0)	0.99
	1548	Variant 2.1 (0.6, 3.6)		

SNP: Single Nucleotide Polymorphism; N: Number of observations; IQR: Inter-quartile range; P: p-value of the interaction term; Ancestral: major frequency allele homozygotes; Variant: minor frequency allele homozygotes and heterozygotes.

*To compute effect estimates from longitudinal models, the time term was fixed at zero.

**Table 3:** **Adjusted changes in pulse pressure (mmHg) with an IQR (20 μg/g) increase in patella lead levels**

SNP	N	Ancestral vs. Variant	Interaction term	
		β (95% CI)*	β (95% CI)*	P
***Bsm1***	811	Ancestral 0.0 (-1.6, 1.5)	1.9 (0.1, 3.8)	0.04
	1619	Variant 1.9 (0.5, 3.2)		
***Taq1***	822	Ancestral -0.1 (-1.6, 1.4)	2.0 (0.2, 3.8)	0.03
	1753	Variant 2.0 (0.7, 3.3)		
***Apa1***	789	Ancestral -0.1 (-1.8, 1.7)	1.7 (-0.3, 3.6)	0.09
	1781	Variant 1.6 (0.4, 2.8)		
***Fok1***	965	Ancestral 1.4 (-0.1, 2.9)	0.3 (-1.5, 2.2)	0.72
	1545	Variant 1.7 (0.3, 3.1)		

SNP: Single Nucleotide Polymorphism; N: Number of observations; IQR: Inter-quartile range; P: p-value of the interaction term; Ancestral: major frequency allele homozygotes; Variant: minor frequency allele homozygotes and heterozygotes.

*To compute effect estimates from longitudinal models, the time term was fixed at zero.

**Figure 1:**
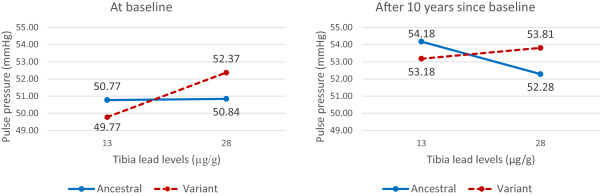
**The predicted values of pulse pressure from the linear mixed model with the continuous tibia lead variable using the tibia bone lead values at the 25** ^**th**^ **percentile and the 75** ^**th**^ **percentile of the distribution at baseline (left) and after 10 years (right) for the ancestral type (solid line) and variant (dashed line) of** ***VDR Bsm1*** **, with all other covariates held constant at the mean (continuous variables: age at baseline (65 yrs), BMI (28 kg/m** ^**2**^ **), and calcium intake from food (800 mg/day)) or zero (categorical variables: race (white), smoking (non-smoker), alcohol intake (less than two drinks/day), diabetes status (no), family history of hypertension (no), and education (completed high school)).**

In Figure 1, we shows the predicted values of pulse pressure from the model with the continuous tibia lead variable by *Bsm1* genotype at baseline (time = 0) and after ten years (time = 10). At baseline, as tibia bone lead level increased from 13 μg/g (25^th^ percentile) to 28 μg/g (75^th^ percentile), pulse pressure increased by 0.07 mmHg (an IQR increase from 50.68 mmHg to 50.75 mmHg) among subjects with *Bsm1* ancestral type, while pulse pressure increased by 2.6 mmHg (from 49.68 mmHg to 52.28 mmHg) among subjects with *Bsm1* variant type. After ten years, the marginal association (when the two lines were combined ignoring the *Bsm1* genotype) between tibia bone lead levels and pulse pressure became weaker. After 10 years of follow-up, as tibia bone lead level increased from 13 μg/g (25^th^ percentile) to 28 μg/g (75^th^ percentile), pulse pressure decreased by 1.9 mmHg (from 54.09 mmHg to 52.19 mmHg) among *Bsm1* ancestral type while pulse pressure increased by 0.63 mmHg (from 53.09 mmHg to 53.72 mmHg) among *Bsm1* variant type for the IQR change in tibia lead levels. In spite of the changing association between bone lead levels and pulse pressure over time, the effect modifications by *VDR Bsm1* and *Taq1* genotypes on the association between bone lead levels and pulse pressure persists over time.

We also conducted cross-sectional analyses using baseline data as a sensitivity analysis. The same covariates except time-since-baseline and its interaction with lead exposure levels were examined. Additional sensitivity analyses were done in order to investigate other possible confounders. Verifying analytical consistency, the interaction effect of bone lead levels and the *VDR* genotype on pulse pressure were examined by (i) adding the square of baseline age, (ii) adding sodium (Na) and potassium (K) intake, (iii) adding heart rate (sitting, beats/min), (iv) adding triglyceride level, (v) adding high-density lipoprotein (HDL) and total cholesterol-to-HDL ratio, (vi) adding total calories (kcal/day) and physical activity (expended (fast walk adjusted), kcal/week), (vii) replacing BMI with height and waist circumference, (viii) replacing two alcohol drinks per day with grams per day of alcohol, (ix) replacing smoking in pack-years with current smoker or not, (x) ignoring calcium intake (obtained from a food frequency questionnaire), (xi) ignoring observations of subjects who are taking blood pressure control medication, and (xii) separating the blood pressure control medication variable into two variables: a calcium channel blocker and others.

We found slightly larger beta values in the cross-sectional baseline analyses (Additional file 1: Table S2). The magnitudes of the beta estimates slightly varied by different covariate sets but the conclusions were consistent (data not shown).

## Discussion

Using longitudinal observations, we found a stronger association between cumulative bone lead levels and pulse pressure in participants with at least one minor frequency allele on *Bsm1* or *Taq1*. The results were consistent for tibia and patella bone lead levels. Our results were robust to cardiovascular risk related confounding factors in the sensitivity analyses. To our knowledge, this is the first study showing the interplay of *VDR* genetic polymorphisms and cumulative lead exposure levels on a CVD subclinical measure in the longitudinal setting.

Pulse pressure is a marker of arterial stiffness, and elevated pulse pressure is a risk factor of CVD. Pulse pressure has been demonstrated as an independent predictor of long-term cardiovascular mortality. A 10 mmHg increase in pulse pressure has been associated with a 10 to 25% increase in risk for CVD related mortality among older adults including coronary heart disease, congestive heart failure, and cerebrovascular disease [29–31]. In the present study, we found that individuals with at least one *VDR Bsm1* variant had a 2.5 mmHg greater pulse pressure in relation to every 15 μg/g increase in cumulative (tibia) lead exposure. We interpret this suggesting that individuals with *VDR Bsm1* at least one variant may have 2.5% to 6% greater risks for CVD mortality for every 15 μg/g increase in cumulative lead exposure.

The two genetic polymorphisms, that we found to interact with the cumulative lead exposure, are common SNPs with minor allele frequency over 0.4 in American Caucasians (Additional file 1: Table S1). Over 65% of European descendants have at least one copy of these genetic polymorphisms, based on HapMap Phase 3 European ancestry samples [32], suggesting that over 65% of European descendants may be susceptible to cumulative lead exposure related elevation in pulse pressure. Thus, the lead by *VDR* interaction found in this study may explain some portion of CVD events and mortality in the elderly.

Underlying biological mechanisms, by which the *VDR* genetic polymorphisms may modify the effect of lead on cardiovascular disease, are not well understood. Individuals with *VDR* genetic variants may have higher body burdens of lead, suggesting that the *VDR* gene may modify the toxicokinetics of lead [12, 24, 33, 34]. In a study of Korean lead workers, blood lead levels and tibia bone lead levels were significantly higher in participants with the *VDR Bsm1* minor frequency allele [35]. Another study of former organolead manufacturing workers in the eastern United States suggested that the *VDR Bsm1* variant influences lead uptake and subsequent release of lead from bone [16]. The interaction between the VDR and lead can be explained by calcium metabolism. The VDR plays a crucial role in calcium absorption and metabolism, which is shared by lead from its divalent cation characteristic. Calcium deficiency was demonstrated to increase lead absorption in the gastrointestinal tract in chicks [36, 37]. Ingestion of lead inhibits the effect of vitamin D and its metabolites on intestinal calcium transport in rats [38]. In addition, associations between high bone and blood lead levels and hypertension were also found among subjects with low dietary calcium intake in the NAS [4].

An interaction between the *VDR* gene and lead was also found to be involved in diverse disease development processes. In a study of the US general population, adults aged 60 years and older with the *VDR* rs2239185–rs731236 (*Taq1*) CC haplotype showed a negative association between blood lead and serum homocysteine, a risk factor of CVD and neurodegenerative disease, while those with the *VDR* rs2239185–rs731236 CT or TT showed a positive association [23]. In the same study, adults aged 20 to 59 years of age who had *VDR* rs2239185–rs731236 CC or TT haplotypes, showed significant decline in cognitive function with increased blood lead concentration while those CT haplotype did not show significant decline. In a study of lead and creatinine among Korean lead workers, the *VDR Bsm1* genotype was also found to modify the association between tibia bone lead and renal function as assessed by serum creatinine level and creatinine clearance [24]. The lead workers with at least one minor frequency allele on *VDR Bsm1* showed worse renal function with higher lead exposure levels. Another study of the Korean lead workers demonstrated an effect modification of the *VDR Bsm1* genotype on the association between lead exposure and blood pressure [39]. Among lead workers with the *VDR Bsm1* variant, SBP were 2.7-3.7 mmHg higher and prevalence of hypertension was higher (OR =2.1). Thus, our results and those of others support the concept of interaction between the *VDR* genotypes and lead.

We observed a significant negative interaction between time-since-baseline and tibia bone lead levels in the longitudinal analyses. The negative interaction can be interpreted in two different ways: the association between tibia bone lead and pulse pressure may decrease during the follow-up or the association between time-since-baseline and pulse pressure may decrease with higher lead exposure levels. A possible explanation for the decreasing effect of cumulative lead levels on pulse pressure during the follow-up is that people who had developed health related problems or diseases were more likely to drop out during follow-up [40]. Hence, the participants who stayed longer in the study may be healthier than those who dropped out. Another possible explanation is that other atherosclerotic risk factors became more pronounced as the study participants get older. As a result, the association between lead exposure and pulse pressure may seem attenuated over time. Alternatively, we have already reported that bone lead concentrations are falling in these participants, and more quickly for patella lead [41]. This suggests that the weaker associations between the baseline bone lead levels and the follow-up pulse pressures could reflect the lower exposure at later follow-up visits. In spite of the changing association between bone lead levels and pulse pressure over time, our main interest, the effect modifications by *VDR Bsm1* and *Taq1* genotypes on the association between bone lead levels and pulse pressure were consistent over time (Figure 1).

We see consistent results with regard to *Bsm1* and *Taq1*. These SNPs are closely located to each other in the *VDR* gene (distance between *Bsm1* and *Taq1* = 1 kilo base pairs, size of the *VDR* gene = 63 kilo base pairs). The two SNPs are in high linkage disequilibrium (r^2^ = 0.92). Among participants with at least one minor frequency allele on *Bsm1*, 98% had at least one minor frequency allele on *Taq1*. This explains why *Bsm1* and *Taq1* show similar effect modification signals. And this consistent results support that our finding is less likely to be a false positive from genotyping errors on *Bsm1* or *Taq1*. In the *VDR* gene, *Bsm1* is located in intron8, and *Taq1* is located in exon9. Genetic polymorphisms in intron regions, where splice enhances or silencers bind, can have an effect on alternative splicing [42]. More than 60% of the alternatively spliced variants in humans results in changes in the protein structure which may result in conformational changes [43]. However, whether these SNPs, *Bsm1* and *Taq1*, are the functional polymorphism with an effect on the structure of vitamin D receptor or on the affinity of the receptor, or whether they are in high linkage disequilibrium with some other functional SNPs is unclear. It should be further investigated in animal studies or *in vitro* studies.

The strengths of the current study include reliable bone lead measurements and extensive follow-ups up to 20 years (median follow-up of 9 years and median number of follow-up examinations of 4). The repeated measurements in pulse pressure and covariates increase statistical power to detect the gene by environment interactions. However, the NAS is an older cohort of predominantly white male participants. Hence, the findings may not be generalizable to women, younger individuals, and other ethnicities.

## Conclusion

Lead toxicity is found in almost every systems in the body. Over 90% of the total body lead burden in adults is accumulated in the bone and only about 1% is found in the blood [44]. Even though blood lead levels have been gradually reduced since the phase-out of leaded gasoline in the 1970s, cumulative lead levels are still substantial in the elderly and associated with diverse diseases including cognitive function decline and hypertension [2, 45]. Our finding suggests that subjects with the minor frequency alleles of *VDR Bsm1* or *Taq1* may be more susceptible to cumulative lead exposure-related elevated pulse pressure. If the VDR gene is involved in the association between lead and pulse pressure, calcium metabolism may play an important role in lead toxicity in CVD. The observed interaction between cumulative lead levels and the VDR Bsm1 or Taq1 persists over time during the follow-up. This implies that the elderly experience adverse effects from their early lead exposure, even though current ambient lead levels are low. Our findings suggest the importance of restricting early exposure to lead to avoid its persistent adverse health effects in the subjects’ later life.

## Electronic supplementary material

Additional file 1: Table S1: Characteristics of genetic markers in *VDR* gene. **Table S2.** Adjusted changes in pulse pressure (mmHg) with at least one minor allele in *VDR* gene per IQR increase in bone lead marker using baseline data. **Table S3.** Adjusted changes in pulse pressure (mmHg) with an IQR (3μg/dL) increase in blood lead levels. (DOCX 22 KB)

## Abbreviations

BMI: Body mass index
CVD: Cardiovascular diseases
DBP: Diastolic blood pressure
HDL: High-density lipoprotein
IQR: Interquartile range
KXRF: K-x-ray fluorescence instrument
NAS: Normative aging study
NTP: National toxicology program
SBP: Systolic blood pressure
SNP: Single nucleotide polymorphism
VDR: Vitamin D receptor.
