# Supplementary material for: Effect modification by vitamin D receptor genetic polymorphisms in the association between cumulative lead exposure and pulse pressure: a longitudinal study
Source: Environ Health. 2015 Jan 13;14:5. doi: 10.1186/1476-069X-14-5 (PMC4417283; doi:10.1186/1476-069X-14-5)
Supplement: Supplementary file 1 — Additional file 1: Table S1: Characteristics of genetic markers in VDR gene. Table S2. Adjusted changes in pulse pressure (mmHg) with at least one minor allele in VDR gene per IQR increase in bone lead marker using baseline data. Table S3. Adjusted changes in pulse pressure (mmHg) with an IQR (3μg/dL) increase in blood lead levels. (DOCX 22 KB) [file 12940_2014_841_MOESM1_ESM.docx]

Additional file

| **Table S1. Characteristics of genetic markers in *VDR* gene.** | | | | | | | |
| --- | --- | --- | --- | --- | --- | --- | --- |
| **SNP** | **Missing** | **Number of**  **minor frequency allele** | |  | **MAF** | **HWE** | **HWE** |
|  |  | **0** | **1 or 2** | |  | **Statistics** | **P-value** |
| *Bsm1* | 47 (6%) | 238 (33%) | 442 (61%) | | 0.42 | 1.22 | 0.27 |
| *Taq1* | 4 (1%) | 242 (33%) | 481 (66%) | | 0.41 | 0.76 | 0.38 |
| *Apa1* | 7 (1%) | 211 (29%) | 509 (70%) | | 0.45 | 0.64 | 0.42 |
| *Fok1* | 22 (3%) | 274 (38%) | 432 (59%) | | 0.37 | 0.25 | 0.62 |
| SNP: Single Nucleotide Polymorphism; MAF: Minor Allele Frequency; HWE: Hardy-Weinberg Equilibrium. | | | | | | | |

| **Table S2. Adjusted changes in pulse pressure (mmHg) with at least one minor allele in *VDR* gene per IQR increase in bone lead marker using baseline data.** | | | | | | | | | |
| --- | --- | --- | --- | --- | --- | --- | --- | --- | --- |
|  | **Tibia lead IQR = 15µg/g** | | | | | **Patella lead IQR = 20µg/g** | | | |
|  |  | | **Ancestral vs. variant** | **Interaction term** | | **N** | **Ancestral vs. variant** | **Interaction term** | |
| **SNP** | | **N** | **β (95% CI)** | **β (95% CI)** | **P** |  | **β (95% CI)** | **β (95% CI)** | **P** |
| ***Bsm1*** | 612 | | Ancestral -0.5 (-2.7, 1.7) | 3.0 ( 0.3, 5.8) | 0.03 | 608 | Ancestral 0.5 (-1.4, 2.4) | 2.0 (-0.4, 4.4) | 0.10 |
|  |  |  | Variant 2.6 ( 0.9, 4.2) |  |  |  | Variant 1.5 (-0.1, 3.1) |  |  |
| ***Taq1*** | 649 | | Ancestral -0.1 (-2.3, 2.1) | 2.4 (-0.3, 5.1) | 0.08 | 645 | Ancestral -0.6 (-2.4, 1.2) | 2.1 (-0.2, 4.5) | 0.07 |
|  |  |  | Variant 2.3 ( 0.7, 3.9) |  |  |  | Variant 1.6 ( 0.0, 3.1) |  |  |
| ***Apa1*** | 647 | | Ancestral 0.9 (-1.5, 3.3) | 0.7 (-2.0, 3.5) | 0.60 | 603 | Ancestral -0.6 (-2.8, 1.6) | 1.6 (-0.9, 4.2) | 0.20 |
|  |  |  | Variant 1.6 ( 0.1, 3.2) |  |  |  | Variant 1.1 (-0.4, 2.5) |  |  |
| ***Fok1*** | 634 | | Ancestral 2.1 ( 0.1, 4.1) | -0.9 (-3.5, 1.7) | 0.51 | 630 | Ancestral 1.1 (-0.7, 2.9) | -0.5 (-2.8, 1.9) | 0.71 |
|  |  |  | Variant 1.2 (-0.5, 3.0) |  |  |  | Variant 0.6 (-1.0, 2.3) |  |  |
| SNP: Single Nucleotide Polymorphism; N: Number of observations; IQR: Inter-quartile range; P: p-value of the interaction term; Ancestral: major frequency allele homozygotes; Variant: minor frequency allele homozygotes and heterozygotes. | | | | | | | | | |

| **Table S3. Adjusted changes in pulse pressure (mmHg) with an IQR (3µg/dL) increase in blood lead levels.** | | | | |
| --- | --- | --- | --- | --- |
| **SNP** | **N** | **Ancestral vs. variant** | **Interaction term** | |
|  |  | **β (95% CI)*** | **β (95% CI)*** | **P** |
| ***Bsm1*** | 686 | Ancestral 0.4 (-0.6, 1.5) | -0.6 (-2.0, 0.7) | 0.34 |
|  | 1317 | Variant -0.2 (-1.2, 0.8) |  |  |
| ***Taq1*** | 695 | Ancestral 0.5 (-0.5, 1.6) | -0.5 (-1.8, 0.7) | 0.42 |
|  | 1433 | Variant 0.01 (-0.9, 0.9) |  |  |
| ***Apa1*** | 636 | Ancestral -0.5 (-1.8, 0.8) | 0.9 (-0.5, 2.3) | 0.21 |
|  | 1485 | Variant 0.4 (-0.4, 1.2) |  |  |
| ***Fok1*** | 801 | Ancestral 0.4 (-0.6, 1.5) | -0.3 (-1.6, 1.0) | 0.63 |
|  | 1269 | Variant 0.1 (-0.8, 1.0) |  |  |
| SNP: Single Nucleotide Polymorphism; N: Number of observations; IQR: Inter-quartile range; P: p-value of the interaction term; Ancestral: major frequency allele homozygotes; Variant: minor frequency allele homozygotes and heterozygotes | | | | |
| *To compute effect estimates from longitudinal models, the time term was fixed at zero. | | | | |
